# Associations Between Centre of Pressure Variables and Performance Penalties in Acrobatic Gymnastics—Feasibility Study

**DOI:** 10.3390/jfmk11030341

**Published:** 2026-08-30

**Authors:** Joana Barreto, Bruno Ramalho, João Abrantes, Sofia Fonseca, Pedro Aleixo

**Affiliations:** 1 Center for Sport, Physical Education, Exercise and Health (CIDEFES), Universidade Lusófona, 1749-024 Lisbon, Portugal; p6609@ulusofona.pt (J.B.); p2546@ulusofona.pt (S.F.); 2 Faculdade de Educação Física e Desporto, Universidade Lusófona, 1749-024 Lisbon, Portugal; brunoramalho060@gmail.com; 3 Human Environment and Interactions Lab (HEI-Lab), Universidade Lusófona, 1749-024 Lisbon, Portugal; joao.mcs.abrantes@ulusofona.pt; 4 Department of Mathematics, Instituto Superior de Engenharia de Lisboa (ISEL), Instituto Politécnico de Lisboa, 1959-007 Lisbon, Portugal

**Keywords:** acrobatic gymnasts, centre of pressure, judges’ penalties, postural control, postural stability, transitional pyramid

## Abstract

**Objectives:** Postural stability is a key component of Acrobatic Gymnastics performance and can be assessed using centre of pressure (COP) variables. This feasibility study provides the first analysis of postural stability during transitional pyramids in Acrobatic Gymnastics by investigating the associations between COP variables and judges’ penalties for body segment positions and postural instability across different phases. Moreover, it also examines the role of each foot in postural stability during performance. **Methods**: Eight acrobatic gymnasts (6 females and 2 males; 4 base and 4 top gymnasts) completed five repetitions of a transitional pyramid, comprising a total of twenty repetitions. However, only eighteen repetitions were eligible for analysis. COP variables (amplitude, peak velocity, and ellipse length, width and area) were extracted during the three phases of the pyramid, and judges’ penalties were obtained from the evaluation of two international acrobatic judges. **Results**: Moderate associations were identified between COP variables and judges’ penalties (*p* < 0.05). Phase 1 yielded consistently lower values for COP variables compared to Phases 2 and 3 (*p* < 0.05). Asymmetries were also observed for selected COP variables, with the left foot exhibiting higher values compared to the right foot (*p* < 0.05). **Conclusions**: The findings of this study support the applicability of the proposed approach and data analysis procedures for future large-scale interventions. Different patterns of associations were observed between COP variables and judges’ penalties across the three phases of the transitional pyramid. Asymmetries were shown between right and left foot.

## 1. Introduction

In Acrobatic Gymnastics, base gymnasts support top gymnasts during transitional pyramids where the top gymnast can move from one static position to another. According to the Acrobatic Gymnastics Code of Points (AGCP), pairs must perform at least five balance elements during a balance exercise [1]. Besides difficulty and artistry, execution, i.e., the quality of technical performance, is evaluated by four to six judges and includes penalties related to the postural stability during balance elements and correctness of body line and shape.

Postural stability can be defined as the ability to maintain or adjust body position through the effective use of motor control mechanisms [2,3]. It presents two forms, i.e., static and dynamic postural stability [4]. Therefore, it is a fundamental aspect of motor control for a successful performance in diverse sports, namely during static and dynamic elements in gymnastics [5,6,7,8,9].

In sports with a high reliance on postural stability, i.e., archery and shooting, findings suggest a relation between postural stability and centre of pressure (COP) data, with superior performance associated with reduced COP displacement [10,11,12], as well as a smaller COP ellipse area [11]. In Acrobatic Gymnastics, the COP path length yields a moderate to very large correlation with technical penalties, with minor penalties applied to acrobatic pyramids with a lower COP path length [6]. A large to near perfect correlation between COP displacement and performance was observed even for acrobatic pyramids with different difficulty values, receiving higher judges’ scores when the COP had a lower displacement [7]. Additionally, COP displacement was greater in the antero-posterior (AP) direction compared to the medio-lateral (ML) direction for the three pyramids. Despite these studies, the literature review revealed a clear lack of studies in this field of research.

**Figure 1:**
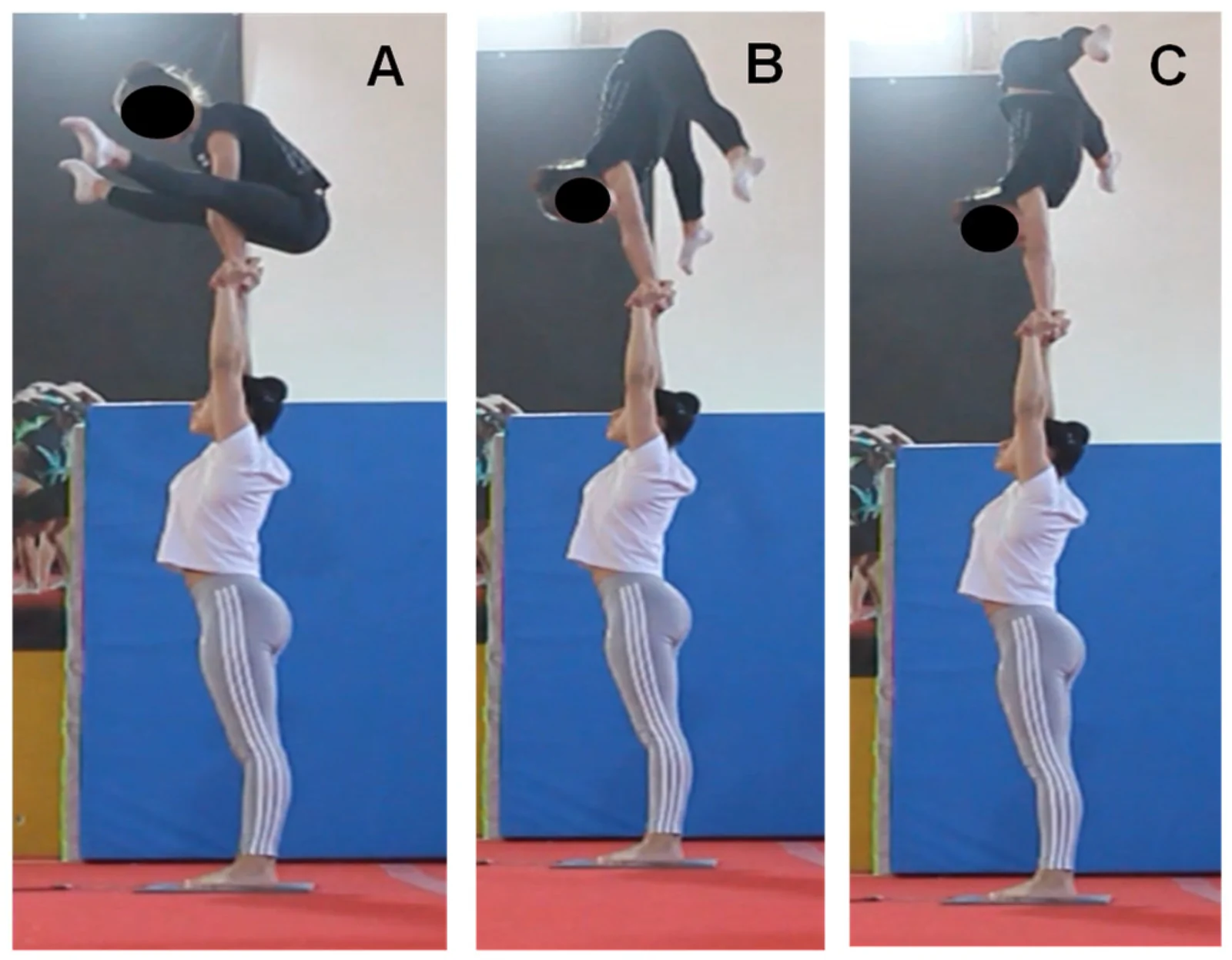
Task performed for analysis: transitional pyramid where the base gymnast supports the top gymnast by the hands; top gymnast initiates the task in a straddle hold position for one second (**A**) and transits (**B**) to a handstand position for three seconds (**C**).

To date, no study has specifically addressed the analysis of transitional pyramids (e.g., Figure 1). Furthermore, the literature reveals inconsistencies in the selection of COP parameters and methodological approaches, limiting the possibility to draw conclusions and propose guidelines. Consequently, questions arise regarding the potential relationship between COP parameters and judges’ penalties across the different phases of transitional pyramids. This study addresses this gap in the literature by providing a comprehensive analysis of postural stability in Acrobatic Gymnastics, with a novel focus on transitional pyramids. In transitional pyramids, the top and base gymnasts can transit between static positions, increasing the level of complexity and leading gymnasts to develop functional strategies related to postural stability and motor control. In accordance, the AGCP rewards transitional pyramids with higher difficulty scores. It is important to acknowledge that COP measures and judges’ evaluations reflect different dimensions of performance. COP variables quantify the interaction between the gymnast and the support surface, capturing the continuous postural adjustments occurring at the base of the kinematic chain. In contrast, judges primarily evaluate the visible outcome of these control processes, namely body alignment, movement quality, and apparent stability. Consequently, compensatory mechanisms occurring at the feet and lower limbs may not always be directly observable, as their effects can be attenuated through the kinematic chain, allowing the gymnast to maintain an apparently stable posture. Understanding this distinction is essential when interpreting potential associations between COP variables and judges’ penalties. By integrating the analysis of the different phases and relationship between biomechanical and technical performance variables, it contributes to the development of evidence-based guidelines for training and evaluation in this sport. Therefore, this study aimed to assess the feasibility of the proposed approach by evaluating the suitability and practicality of the assessment protocol and data analysis procedures for future large-scale investigations. Specifically, this feasibility study aimed: (a) to investigate associations between COP data and judges’ penalties for body segment position and postural instability across different phases of a transitional pyramid [13]; and (b) to examine the role of each foot in postural stability during performance. It was hypothesized that: (a) COP data and judges’ penalties would be positively associated for all phases of the pyramid; (b) differences would be observed for all variables during the different phases of the pyramid; and (c) COP data of each foot would differ, suggesting that the right and left foot have different roles in maintaining the stability of the pyramid.

## 2. Materials and Methods

### 2.1. Participants

The inclusion criteria for participants were (a) being affiliated at Portuguese Gymnastics Federation, (b) participating in national and international competitions in 2024–2025, (c) and being free from musculoskeletal injuries in the last three months. Also, participants should be able to perform the task in the study autonomously, ensuring their safety during the study. Eight acrobatic gymnasts (6 females and 2 males; 4 base gymnasts: 20.25 ± 5.26, and 4 top gymnasts: 12.75 ± 1.48 years old) participated in the study. They had 6.56 ± 3.97 years of experience, trained 3 h a day at least 4 times a week and met all the inclusion criteria. One female pair belonged to the pre-youth category, another to the youth category, and the third to the senior category. The male pair belonged to the junior elite category.

The study was approved by the Ethical Committee of the Faculdade de Educação Física e Desporto, Universidade Lusófona, Lisboa, Portugal (Ref. No. F2526C), and written informed consent was obtained from each participant/guardian prior to data collection. All procedures complied with the Declaration of Helsinki.

### 2.2. Protocol

The data collection session followed this sequence: (a) collection of anthropometric and demographic data; (b) warm-up; and (c) data collection. This session took place in the gymnasium hall where the athletes usually train.

Participants performed a typical and usual warm-up that included cardiorespiratory, flexibility and activation exercises. Balance exercises were also included, and the warm-up took approximately 20 min. The transitional pyramid under study is illustrated in Figure 1. It starts with the base gymnast supporting by the hands the top gymnast, who maintains a straddle position for one second (Figure 1A) and transits (Figure 1B) to a handstand position (Figure 1C) that is kept for three seconds. This task was selected to ensure the analysis of two different static positions (Figure 1A,C) and one dynamic transitional phase (Figure 1B), as well as representativeness of various categories of elite level.

Participants practiced the balance task for five minutes to get used to the platform. When participants were ready, they were required to execute five successful repetitions (i.e., no falls, no deviations from the platform, compliance with each phase duration) of the balance task on the pressure platform. If a repetition was not successful, participants were asked to repeat it, until five successful repetitions were recorded. A rest interval of 2 min was allowed between repetitions to avoid fatigue. A total of twenty repetitions were recorded; however, two repetitions were excluded from the analysis due to data acquisition errors on the pressure platform. A total of eighteen eligible repetitions were included in the analysis.

A pressure platform (RSscan International, Olen, Belgium; 1000 × 500 mm; 50 Hz) was used to collect COP data. In addition, the athletes’ performances were recorded using a video camera (CANON EOS R6 Mark II, Canon Inc., Tokyo, Japan; 25 Hz) for subsequent evaluation by the judges. The camera was synchronized with the pressure platform to identify the three movement phases and was placed laterally to the platform at 5 m.

### 2.3. Judges’ Evaluation

Two international Acrobatic Gymnastics judges from Portugal were asked to evaluate technical performance, according to the AGCP [1]. The judges received the videos from the performances together with a document adapted from the AGCP [1]. Both judges evaluated all the repetitions in respect to the penalties (points) relative to postural instability and body segment position, separately, for each of the three phases of the balance task (e.g., 0.1 for small, 0.2–0.3 for significant, 0.5 for serious faults and 1.0 for falls). To assess scoring consistency, inter-rater reliability was calculating using a two-way mixed-effects model with average measures (ICC3,k). Consequently, the final penalty scores used in all statistical analysis represented the average of the deductions assigned by the two raters.

### 2.4. COP Variables Data Processing

**Figure 2:**
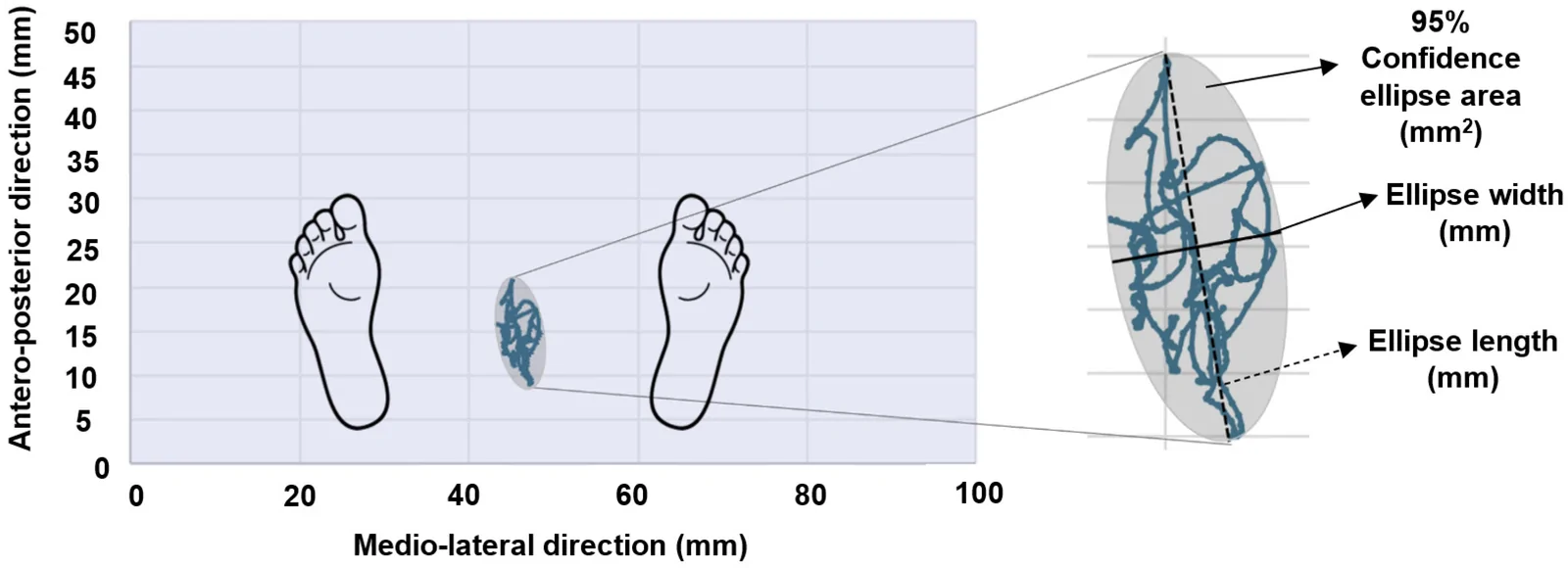
Example of COP trajectory and 95% confidence ellipse calculation during one repetition for phase 3. **Left**: COP displacement on the platform relative to feet position with medio-lateral and antero-posterior axis (mm). **Right**: amplified view of the calculated ellipse with labels for width (mm, full line), length (mm, dashed line) and area (mm^2^, shaded area).

COP variables were obtained from the Footscan 7 software outputs. Derived variables, such as peak COP speed, were calculated from the 50 Hz positional data. No additional digital filtering (e.g., Butterworth filter) was applied prior to statistical analysis. For the three movement phases, the following COP variables were calculated (Figure 2): (a) AP and ML amplitude (mm), defined as the difference between the maximum and minimum COP values along each axis, i.e., peak-to-peak displacement [14]; (b) AP and ML peak velocity (mm/s); velocity values calculated from COP position data using the finite-difference method [14]; (c) 95% confidence ellipse area (mm^2^) of the COP displacement [15]; (d) length and width (mm) of the 95% confidence ellipse area [16]. These variables were calculated for the overall COP, as well as for the COP of each foot.

### 2.5. Statistical Analysis

Statistical analyses were conducted using JASP (Jasp Team 2023, version 0.17.3, available at https://jasp-stats.org/download/ (accessed on 9 March 2026). Given the exploratory nature of this feasibility study and the small sample size (n = 8), parametric tests were applied under the assumption of data linearity, considering the known robustness of these procedures to moderate distributional variations and to ensure comparability with previous research in Acrobatic Gymnastics. The Pearson correlation was applied to measure and test the correlation between COP variables and judges’ penalties, for each movement phase, considering 0.00–0.10 as negligible correlation, 0.10–0.39 as weak correlation, 0.40–0.69 as moderate correlation, 0.70–0.89 as strong correlation and 0.90–1.00 as very strong correlation [17]. Additionally, these variables were compared between each movement phase using ANOVA tests. When statistically significant differences were found, the respective post-hoc tests were applied. Moreover, in each phase, COP variables of right and left foot were compared using the paired *t*-test [18].

## 3. Results

### 3.1. Descriptive Data

Moderate-to-good agreement inter-rater agreement [19] was observed for both body segment position penalties (ICC3,k = 0.701, 95% CI: [0.485, 0.827]) and postural instability penalties (ICC3,k = 0.729, 95% CI: [0.532, 0.843]).

Table 1 presents the results of COP variables and judges’ penalties across each movement phase.

### 3.2. Correlations Between COP Variables and Judges’ Penalties

Regarding overall COP (as showed in Appendix A.1), results showed moderate correlations between ellipse width and postural instability (r = 0.47, *p* < 0.05) and body segment position penalties in phase 3 (r = 0.58, *p* < 0.01). No statistically significant correlations were found for the other COP variables and judges’ penalties.

For the right foot, moderate correlations were found between ML amplitude and postural instability penalties in phase 2 (r = 0.50, *p* < 0.05) and between ML peak velocity and body segment position penalties in phase 1 (r = −0.49, *p* < 0.05). No statistically significant correlations were found for the other COP variables and judges’ penalties.

For the left foot, moderate correlations were found between AP peak velocity and postural instability penalties in phase 2 (r = 0.51, *p* < 0.05) and between ellipse area and postural instability penalties in phase 1 (r = −0.51, *p* < 0.05). No statistically significant correlations were found for the other COP variables and judges’ penalties.

### 3.3. Comparison Between Phases Regarding COP Variables

As showed in Table 2, overall COP differed significantly between the three phases with respect to ML amplitude (*p* < 0.001), AP amplitude (*p* < 0.001), AP peak velocity (*p* < 0.05), ellipse length (*p* < 0.001), ellipse width (*p* < 0.001), and ellipse area (*p* < 0.001).

Regarding the COP of the left and right foot, differences were found between the three phases, namely for AP amplitude and ellipse length (*p* < 0.001). Also, differences between the three phases were found for ML amplitude (*p* < 0.01) of the right foot. No differences were found for the remaining COP variables (Appendix A.2).

The post-hoc tests (Table 3) revealed differences for both feet ML amplitude and AP amplitude between phases 1 and 2 (*p* < 0.001) and 1 and 3 (*p* < 0.001), ellipse length, ellipse width and ellipse area between phases 1 and 2 (*p* < 0.001) and 1 and 3 (*p* < 0.001). Differences were yielded for ML amplitude right between phases 1 and 2 and 1 and 3 (*p* < 0.05). Also, significant differences were shown for AP amplitude left between phases 1 and 2 and 1 and 3 (*p* < 0.001) and AP amplitude right between phases 1 and 2 (*p* < 0.001) and 1 and 3 (*p* < 0.001). Finally, differences were observed for ellipse length left and right between phases 1 and 2 (*p* < 0.001) and 1 and 3 (*p* < 0.001). Additionally, differences were revealed for ellipse length of the right foot between phases 2 and 3 (*p* < 0.05) with greater values for phase 3. No other significant differences were observed between phases 2 and 3 across the remaining COP variables (Appendix A.3). Across all COP variables with significant between-phase differences, phases 2 and 3 consistently presented higher values than phase 1 (Table 1).

### 3.4. Comparison Between Phases Regarding Judges’ Penalties

Regarding judges’ penalties, differences were found between the three phases for postural instability penalties (F = 65.55, *p* < 0.001), but not for body segment position penalties. The post-hoc tests revealed differences for the postural instability penalties between phases 1 and 2 (M diff = −0.05, SE = 0.01, *t* = −4.46, *p* < 0.001), phases 1 and 3 (M diff = −0.13, SE = 0.01, *t* = −11.36, *p* < 0.001), and phases 2 and 3 (M diff = −0.08, SE = 0.01, *t* = −6.91, *p* < 0.001).

### 3.5. Comparison Between Right and Left Foot Across the Three Phases

As shown in Table 4, differences were found between the right and left foot for AP amplitude and ellipse length during phases 1, 2 and 3 (*p* < 0.05). Additionally, differences were also observed for ML amplitude and ellipse area during phase 2, and for AP peak velocity during phase 3 (*p* < 0.05). Specifically, higher values were observed for the left foot, except for ML amplitude in phase 2, which was higher for the right foot. No statistically significant differences were found for the other COP variables (Appendix A.4).

## 4. Discussion

This study aimed to assess the feasibility of the proposed approach by evaluating the suitability and practicality of the assessment protocol and data analysis procedures for future large-scale investigations. The assessment protocol was successfully implemented with participants completing all procedures and the pressure platform recordings allowing the extraction of all COP variables across the task phases. These findings supported the suitability of the protocol for characterising postural control during transitional pyramids and demonstrated that the selected variables effectively capture phase-specific and foot-specific COP characteristics. The judges’ assessment protocol was successfully implemented, with both judges able to systematically evaluate all performances according to the AGCP. The data analysis procedures proved to be reproducible, although time consuming. Optimization of the data analysis should be considered prior to future large-scaled interventions. Overall, the findings support the feasibility of the proposed methodology for future studies and provide valuable information for refining the data analysis procedures.

The analysis explored potential phase- and foot-dependent associations between COP variables and judges’ penalties. Across the three phases of the transitional pyramid, different patterns of associations were observed, suggesting that the perceptual relevance of postural stability varies throughout the task phases.

An important consideration when interpreting these findings is that COP variables and judges’ evaluations do not assess the same construct. COP measures reflect the interaction between the gymnast and the support surface and provide information regarding the corrective postural adjustments performed at the feet and lower limbs. Conversely, judges evaluate the visible outcome of these adjustments, focusing on body alignment, movement execution, and apparent stability. From a kinematic chain perspective, perturbations occurring at the feet may be partially compensated by proximal segments, allowing the gymnast to preserve an apparently stable body position. Therefore, substantial COP excursions may coexist with relatively small execution penalties when corrective strategies are effective. This distinction may help explain why some COP variables were only moderately associated with judges’ penalties and why the strength of these associations differed across phases of the transitional pyramid.

Contrary to hypothesis A, which anticipated positive correlations between COP variables and judges’ penalties across all phases, data suggest that greater COP displacements and faster postural adjustments during the straddle position (phase 1) were not necessarily associated with higher penalties. More specifically, the negative correlation between ML peak velocity and body segment position penalties suggests that faster corrective actions may facilitate the maintenance of body alignment and reduce visible execution error. Similarly, the negative correlation between left foot ellipse area and postural instability penalties suggests that stability variability is not necessarily interpreted as reduced performance quality. Since judges rely exclusively on visual observation, these imperceptible adjustments captured by the pressure platform prevent visual execution errors, explaining why a higher ML peak velocity and left foot ellipse area in phase 1 resulted in lower penalties. Previous research in Acrobatic Gymnastics has consistently shown that higher COP displacements are associated with lower performance scores when analysing static pyramids with a duration of seven seconds [6,7], suggesting that greater overall stability contributes to higher-quality performances. This apparent discrepancy may stem from the different dimensions of postural control being assessed. While COP displacement and inter-trial variability reflect overall stability and consistency of performance, variables that we examined may capture the continuous corrective adjustments occurring within a single repetition. Additionally, the first phase of transitional pyramids may not only capture initial stabilization processes, but, most importantly, anticipatory postural adjustments (APAs) that facilitate the forthcoming transition (phase 2). APAs represent feedforward control mechanisms that precede voluntary movements, organizing dynamic joint equilibrium and modulating centre of pressure dynamics to minimize potential postural perturbations [20,21]. In this context, a higher ML peak velocity may reflect a proactive, functional postural strategy to regulate whole-body momentum rather than true mechanical instability [22]. Given the short duration of phase 1, it is unlikely that judges directly perceive these rapid, continuous COP adjustments but instead, judge based on visible kinematic outcomes, reflecting the significant lower postural instability penalties when comparing phase 1 with the remaining phases, but rendering phase 1 the second highest phase for body segment position penalties.

During the transition phase (phase 2), left foot COP ML peak velocity and right foot COP AP amplitude showed positive associations with judges’ instability penalties, which is consistent with previous findings [6,7]. As this phase involves the transition of the top gymnast into a handstand while the base gymnast maintains the support position, temporary misalignments between the gymnasts are expected, necessitating corrective postural adjustments by the base gymnast that are reflected in the COP data. Due to the characteristics and difficulty of this transitional phase, these corrective strategies between base and top gymnasts might be more externally observable and influence judges’ perception of stability. Although the observed correlations are moderate, these objective measures of the COP may provide relevant performance feedback [6].

The handstand position (phase 3) demands postural control strategies to end the transitional movement and maintain a final position for three seconds. A positive association was observed between ellipse width and judges’ penalties, which is more aligned with the findings from previous studies [6,7]. Compared to the remaining phases, this was the phase with superior postural instability and body segment position penalties. With a longer duration and higher difficulty, the strategies to compensate and correct misalignments and oscillations of COP probably become more visually detected by judges, that apply greater penalties, although these adjustments may reflect a functional strategy to succeed in the task (i.e., maintain the position for three seconds without falling). Moreover, associations found in this study indicate that an excessive instability in the final phase may negatively impact performance outcomes.

As in a previous study [6], differences between feet for COP variables were found during static phases, as well as during the transitional phase. These asymmetries may suggest that feet have different and specific roles, related to load support and control, in order to maintain postural stability even in more complex transitional pyramids. This topic warrants further investigation in future research.

The findings obtained in this study highlight the value of combining objective COP measures with judges’ evaluations to improve the understanding of how gymnasts regulate postural instability during transitional pyramids and how these control strategies are reflected in performance assessment. This integrated approach may provide practical information for coaches by identifying phase- and foot-specific postural control demands and support the design of training interventions to optimize performance.

Several limitations are worth acknowledging in this study. Although the gymnasts were all international-level gymnasts, the sample size was relatively small. As all gymnasts belonged to the same team, caution should be exercised when generalising the findings to other populations or competitive levels. The proposed protocol was evaluated using only one transitional pyramid, and it remains uncertain whether similar findings would be obtained in other acrobatic tasks. Also, the pressure platform operated at a frequency of 50 Hz, which although sufficient for COP displacement variables, represents a relatively low acquisition rate for variables such as COP velocity. Finally, a limitation of this study is the sample heterogeneity considering age, body mass and dimensions, and competitive levels across pairs. Differences in these variables may directly influence absolute values of COP variables and judges’ penalties, as body mass and height alter mass distribution and inertial properties in postural control tasks. Technical level varies between competitive categories, which may influence the strategies of COP adjustments. Future studies should incorporate pressure platforms with higher sampling frequencies (≥100 Hz), include more transitional pyramids, and a sample with a larger category-specific cohort to isolate the effects of morphological and technical levels on the variables in study.

## 5. Conclusions

The findings of this feasibility study support the applicability of the proposed protocol and data analysis procedures for future large-scale studies. Although optimization of the data analysis is needed, the methodology is feasible for integrating COP data with judges’ evaluations during transitional pyramids.

This was the first study that investigated associations between COP data and judges’ penalties during transitional pyramids. Across the three phases of the transitional pyramid, different patterns of associations were observed between COP variables and performance variables, suggesting that the perceptual relevance of postural stability varies throughout the task phases. The observed asymmetries between the left and right foot emphasized a potential area for future investigation.

In conclusion, this study demonstrates that COP measures may provide objective information associated with judges’ assessment during transitional pyramids, offering a foundation for future large-scale investigations. Combining these two bodies of data may contribute to better understanding postural control strategies, support evidence-based training interventions and refine performance evaluation in Acrobatic Gymnastics.

## Figures and Tables

**Table 1:** Mean and standard deviation of the COP variables (results of the overall COP as well as of the COP of each foot, considering the three phases) and judges’ penalties.

COP Data	Phase	Mean ± Standard Deviation		
		Both Feet	Right Foot	Left Foot
ML Amplitude (mm)	1	14.92 ± 8.68	9.46 ± 7.10	10.27 ± 8.67
	2	36.55 ± 10.73	19.19 ± 13.06	15.71 ± 9.38
	3	36.83 ± 11.87	19.41 ± 14.82	16.13 ± 10.87
AP Amplitude (mm)	1	19.68 ± 9.66	18.58 ± 8.35	22.83 ± 12.42
	2	60.62 ± 21.68	56.44 ± 21.57	69.03 ± 20.53
	3	61.66 ± 28.39	59.10 ± 29.33	69.23 ± 28.02
ML Peak velocity (mm/s)	1	124.13 ± 104.7	137.30 ± 125.30	147.22 ± 155.50
	2	166.80 ± 79.20	96.56 ± 67.25	80.58 ± 45.37
	3	158.50 ± 59.14	94.36 ± 75.06	85.49 ± 57.26
AP Peak velocity (mm/s)	1	141.41 ± 137.20	225.05 ± 174.03	250.45 ± 170.10
	2	221.90 ± 75.71	266.30 ± 89.64	318.90 ± 94.32
	3	219.00 ± 118.40	258.70 ± 105.50	312.50 ± 138.00
Ellipse length (mm)	1	14.90 ± 8.77	15.40 ± 7.28	18.77 ± 10.37
	2	36.96 ± 10.63	38.96 ± 16.74	45.43 ± 14.96
	3	36.47 ± 11.65	40.64 ± 19.72	47.74 ± 19.98
Ellipse width (mm)	1	18.84 ± 10.08	2.34 ± 4.04	3.82 ± 3.67
	2	67.40 ± 21.07	1.67 ± 0.83	2.10 ± 0.83
	3	61.07 ± 27.71	1.85 ± 1.42	2.24 ± 1.26
Ellipse area (mm^2^)	1	119.41 ± 110.74	38.42 ± 76.56	70.66 ± 100.12
	2	606.75 ± 281.83	55.98 ± 38.53	78.07 ± 44.36
	3	592.91 ± 354.54	73.80 ± 86.86	95.95 ± 82.94
Judges’ penalties	Phase	Mean ± standard deviation		
Postural instability (points)	1	0.01 ± 0.02		
	2	0.06 ± 0.03		
	3	0.15 ± 0.05		
Body segment position (points)	1	0.12 ± 0.14		
	2	0.09 ± 0.05		
	3	0.14 ± 0.07		

**Table 2:** ANOVA statistically significant results for the comparison of the three phases regarding COP variables (considering the overall COP variables as well as the COP variables of each foot). COP variables that present no differences between phases are not presented (all data are presented in Appendix A.2 in Table A2).

COP Data	Phase	Both Feet	Right Foot	Left Foot
		F (2;54)	F (2;54)	F (2;54)
ML Amplitude (mm)	1	29.06 ***	7.99 **	2.95
	2			
	3			
AP Amplitude (mm)	1	27.45 ***	24.05 ***	36.61 ***
	2			
	3			
AP Peak velocity (mm/s)	1	3.45 *	0.57	1.61
	2			
	3			
Ellipse length (mm)	1	26.68 ***	26.33 ***	19.42 ***
	2			
	3			
Ellipse width (mm)	1	25.61 ***	0.327	2.79
	2			
	3			
Ellipse area (mm^2^)	1	21.55 ***	1.10	0.42
	2			
	3			

Note: F: ANOVA F statistic. * *p* < 0.05, ** *p* < 0.01, *** *p* < 0.001.

**Table 3:** Post-hoc pairwise comparison for the comparison of the three phases regarding COP variables showing significant differences between phases, considering the overall COP variables as well as the COP variables of each foot. COP variables that present no differences between phases are not presented (all data are presented in Appendix A.2 in Table A2).

COP Data	Condition	Phases	M Diff	SE	*t*
ML Amplitude (mm)	Both feet	1 vs. 2	−21.63	2.25	−9.64 ***
		1 vs. 3	−21.92	3.64	−6.02 ***
	Right foot	1 vs. 2	−9.73	2.84	−3.43 *
		1 vs. 3	−9.96	3.40	−2.93 *
AP Amplitude (mm)	Both feet	1 vs. 2	−40.94	5.60	−7.31 ***
		1 vs. 3	−41.98	7.26	−5.79 ***
	Right foot	1 vs. 2	−37.86	5.30	−7.14 ***
		1 vs. 3	−40.52	7.50	−5.40 ***
	Left foot	1 vs. 2	−46.21	5.86	−7.89 ***
		1 vs. 3	−46.40	7.05	−6.58 ***
AP Peak velocity (mm/s)	Both feet	1 vs. 2	−80.52	30.17	−2.67 *
Ellipse length (mm)	Both feet	1 vs. 2	−21.18	3.38	−6.27 ***
		1 vs. 3	−21.58	3.38	−6.38 ***
	Right foot	1 vs. 2	−25.24	5.50	−4.59 ***
		1 vs. 3	−32.34	5.45	−5.93 ***
		2 vs. 3	−7.10	2.42	−2.94 *
	Left foot	1 vs. 2	−26.66	4.44	−6.01 ***
		1 vs. 3	−28.98	5.41	−5.35 ***
Ellipse width (mm)	Both feet	1 vs. 2	−41.91	6.79	−6.17 ***
		1 vs. 3	−42.23	6.79	−6.22 ***
Ellipse area (mm^2^)	Both feet	1 vs. 2	−488.60	84.35	−5.79 ***
		1 vs. 3	−469.91	84.35	−5.57 ***

Note: M diff: Mean difference, SE: standard error, *t*: test statistic. * *p* < 0.05, *** *p* < 0.001.

**Table 4:** Results of paired samples *t*-tests comparing right and left foot across the three phases. COP variables that presented no differences between phases are not presented (all data are presented in Appendix A.4 in Table A4).

COP Data	Phase	*t* (17)	*p*	*d*
ML Amplitude (mm)	2	2.24 *	0.04	0.53
AP Amplitude (mm)	1	−2.39 *	0.03	−0.56
	2	−3.86 ***	0.00	−0.91
	3	−3.04 **	0.01	−0.72
AP Peak velocity (mm/s)	3	−2.71 *	0.02	−0.64
Ellipse length (mm)	1	−2.26 *	0.04	−0.53
	2	−2.34 *	0.03	−0.55
	3	−2.94 **	0.01	−0.69
Ellipse area (mm^2^)	2	−2.21 *	0.04	−0.52

Note: *t*: test statistic, *p*: *p*-value, *d*: Cohen’s d effect, * *p* < 0.05, ** *p* < 0.01, *** *p* < 0.001.

## Data Availability

The data supporting the findings of this study are available from the corresponding author upon reasonable request, subject to institutional, ethical, and privacy restrictions.

## Appendix A

### Appendix A.1

**Table A1:** Correlation coefficients between COP data and judges’ penalties for each phase for both feet and for left and right foot separately.

COP Data	Phase	Both Feet		Right Foot		Left Foot	
		Postural Instability Penalties	Body Segment Position Penalties	Postural Instability Penalties	Body Segment Position Penalties	Postural Instability Penalties	Body Segment Position Penalties
ML Amplitude (mm)	1	0.10	−0.06	−0.02	−0.27	0.07	−0.26
	2	0.14	−0.07	0.50 *	0.05	0.25	0.15
	3	0.13	0.23	0.46	0.08	0.34	0.19
AP Amplitude (mm)	1	−0.07	−0.16	−0.16	−0.21	−0.03	−0.06
	2	0.42	−0.24	0.33	−0.41	0.44	−0.10
	3	0.25	−0.08	0.18	−0.17	0.25	0.14
ML Peak velocity (mm/s)	1	0.29	−0.46	0.17	−0.49 *	−0.05	−0.31
	2	0.44	0.30	0.39	0.06	0.29	0.26
	3	−0.01	0.34	0.45	0.12	0.32	0.21
AP Peak velocity (mm/s)	1	0.22	−0.16	0.34	−0.40	0.27	−0.32
	2	0.42	−0.24	0.12	−0.32	0.51 *	0.15
	3	0.45	0.09	0.37	−0.11	0.46	0.22
Ellipse length (mm)	1	−0.02	−0.09	−0.17	−0.24	0.02	−0.23
	2	0.17	0.22	0.41	−0.34	0.47	−0.01
	3	0.31	0.20	0.23	−0.17	0.17	0.12
Ellipse width (mm)	1	−0.26	−0.20	−0.15	−0.43	−0.44	−0.09
	2	0.41	0.35	0.40	−0.22	0.13	−0.03
	3	0.47 *	0.58 **	0.00	−0.03	−0.01	−0.00
Ellipse area (mm^2^)	1	−0.12	−0.17	−0.18	−0.44	−0.51 *	−0.17
	2	0.38	0.44	0.42	−0.33	0.35	−0.04
	3	0.25	0.46	−0.04	−0.03	0.01	0.05

Note: * *p* < 0.05, ** *p* < 0.01.

### Appendix A.2

**Table A2:** ANOVA results for the comparison of the COP data between the three phases for both feet and for left and right foot separately.

COP Data	Phase	Both Feet	Right Foot	Left Foot
		F (2;54)	F (2;54)	F (2;54)
ML Amplitude (mm)	1	29.06 ***	7.99 **	2.95
	2			
	3			
AP Amplitude (mm)	1	27.45 ***	24.05 ***	36.61 ***
	2			
	3			
ML Peak velocity (mm/s)	1	1.57	2.44	3.08
	2			
	3			
AP Peak velocity (mm/s)	1	3.45 *	0.57	1.61
	2			
	3			
Ellipse length (mm)	1	26.68 ***	26.33 ***	19.42 ***
	2			
	3			
Ellipse width (mm)	1	25.61 ***	0.33	2.79
	2			
	3			
Ellipse area (mm^2^)	1	21.55 ***	1.10	0.42
	2			
	3			

Note: F: ANOVA F statistic. * *p* < 0.05, ** *p* < 0.01, *** *p* < 0.001.

### Appendix A.3

**Table A3:** Post-hoc test results between paired phases for the COP data and judges’ penalties for both feet and for left and right foot separately.

		Both Feet							
COP Data	Phases	M Diff			SE			*t*	
ML Amplitude (mm)	1 vs. 2	−21.63			2.25			−9.64 ***	
	1 vs. 3	−21.92			3.64			−6.02 ***	
	2 vs. 3	−0.282			3.79			−0.08	
AP Amplitude (mm)	1 vs. 2	−40.94			5.60			−7.31 ***	
	1 vs. 3	−41.98			7.26			−5.79 ***	
	2 vs. 3	−1.04			6.42			−0.16	
AP Peak velocity (mm/s)	1 vs. 2	−80.52			30.17			−2.67 *	
	1 vs. 3	−77.55			43.68			−1.78	
	2 vs. 3	2.97			28.42			0.10	
Ellipse length (mm)	1 vs. 2	−21.18			3.38			−6.27 ***	
	1 vs. 3	−21.58			3.38			−6.38 ***	
	2 vs. 3	−0.40			3.38			−0.12	
Ellipse width (mm)	1 vs. 2	−41.91			6.79			−6.17 ***	
	1 vs. 3	−42.23			6.79			−6.22 ***	
	2 vs. 3	−0.33			6.79			−0.05	
Ellipse area (mm^2^)	1 vs. 2	−488.60			84.35			−5.79 ***	
	1 vs. 3	−469.91			84.35			−5.57 ***	
	2 vs. 3	18.69			84.35			0.22	
**COP data**	**Phases**	**Right foot**				**Left foot**			
		**M diff**	**SE**	***t***		**M diff**	**SE**		***t***
ML Amplitude (mm)	1 vs. 2	−9.73	2.84	−3.43 *					
	1 vs. 3	−9.96	3.40	−2.93 *					
	2 vs. 3	−0.23	2.15	−0.11					
AP Amplitude (mm)	1 vs. 2	−37.86	5.30	−7.14 ***		−46.21	5.86		−7.89 ***
	1 vs. 3	−40.52	7.50	−5.40 ***		−46.40	7.05		−6.58 ***
	2 vs. 3	−2.66	6.62	−0.40		−0.20	6.89		−0.03 *
Ellipse length (mm)	1 vs. 2	−25.24	5.50	−4.59 ***		−26.66	4.44		−6.01 ***
	1 vs. 3	−32.34	5.45	−5.93 ***		−28.98	5.41		−5.35 ***
	2 vs. 3	−7.10	2.42	−2.94 *		−2.32	5.58		−0.42

Note: M diff: Mean difference, SE: standard error, *t*: test statistic, 1: Phase 1, 2: Phase 2, 3: Phase 3. * *p* < 0.05, *** *p* < 0.001.

### Appendix A.4

**Table A4:** Results of paired samples *t*-tests comparing right and left foot across the three phases for COP data.

COP Data	Phase	*t* (17)	*p*	*d*
ML Amplitude (mm)	1	−0.35	0.73	−0.08
	2	2.24 *	0.04	0.53
	3	1.97	0.07	0.46
AP Amplitude (mm)	1	−2.39 *	0.03	−0.56
	2	−3.86 ***	0.00	−0.91
	3	−3.04 **	0.01	−0.72
ML Peak velocity (mm/s)	1	−0.35	0.73	−0.08
	2	1.71	0.11	0.40
	3	1.23	0.24	0.29
AP Peak velocity (mm/s)	1	−0.82	0.43	−0.19
	2	−1.90	0.07	−0.45
	3	−2.71 *	0.02	−0.64
Ellipse length (mm)	1	−2.26 *	0.04	−0.53
	2	−2.34 *	0.03	−0.55
	3	−2.94 **	0.01	−0.69
Ellipse width (mm)	1	−1.33	0.20	−0.31
	2	−1.89	0.08	−0.45
	3	−1.34	0.20	−0.32
Ellipse area (mm^2^)	1	−1.24	0.23	−0.29
	2	−2.21 *	0.04	−0.52
	3	−1.42	0.18	−0.33

Note: *t*: test statistic, *p*: *p*-value, *d*: Cohen’s D effect, * *p* < 0.05, ** *p* < 0.01, *** *p* < 0.001.
